# Supplementary material for: Minimal residual disease detection by next-generation sequencing of different immunoglobulin gene rearrangements in pediatric B-ALL
Source: Nat Commun. 2023 Nov 17;14:7468. doi: 10.1038/s41467-023-43171-9 (PMC10656538; doi:10.1038/s41467-023-43171-9)
Supplement: Supplementary file 1 — Supplementary Information [file 41467_2023_43171_MOESM1_ESM.pdf]

Supplementary Information

1. Data Quality Evaluation

For the 399 Pre (initial diagnosis) samples and the 734 Post (follow-up) samples, we conducted the following analyses:

a. Number of Input Cells for Sequencing: The number of input cells was calculated based on the amount of DNA used in each experiment, standardized at 6.4 pg per cell. For the Pre samples, the primary objective was to identify dominant clones. To that end, we opted for a smaller sample size dictated by the sampling situation. In contrast, for the Post samples, our aim was to maximize the sensitivity of our detection methods; therefore, we used as many samples as clinically feasible.

b. Size of Sequencing Raw Data: This metric refers to the number of FASTQ reads generated for each sample. Due to our aim of maximizing sensitivity in the Post samples, the raw FASTQ data size for these samples was larger than that for the Pre samples.

c. Q30 Value of the FASTQ Data: The Q30 value serves as a standard measure for evaluating the quality of the base calls during sequencing. The specific information can be found in Supplementary Table 1.

Regarding stability of IGK/IGL markers, the data for both Pre-treatment and Post-treatment samples were analyzed separately to determine the clone counts for IGK and IGL markers. Given the variability in treatment received across different patients, some discrepancies in the quantity of markers may be observed in the raw data. For specific results, please consult Supplementary Table 1.

To statistically assess the data quality and stability, the data related to total cell counts, raw data read counts, raw data Q30 values, IGK counts, and IGL counts underwent quality assessment utilizing the 3-sigma (3-σ) statistical method[1,2,3]. It's noteworthy that the 3-σ principle is based on a normal distribution, where roughly 68% of observations fall within one standard deviation (σ) from the mean, about 95% within two standard deviations, and approximately 99.7% within three standard deviations. In practical applications, especially with limited sample sizes, deviations may occur.

Initially, we performed a normality test on each of the five data sets. As outlined in Supplementary Table 2, all five categories demonstrated high levels of normality with p-values less than 0.0001, thereby confirming a statistically significant normal distribution for each set of data.

Subsequently, we conducted a more in-depth examination of the standard deviations using the 3-sigma (3-σ) test principle, as depicted in Supplementary Figure 1. In both Pre and Post samples, more than 95% of the data fell within the $\mu \pm 2\sigma$ range, and roughly 99% within the $\mu \pm 3\sigma$ range. Although there was a minor deviation from the theoretical 99.7% within the $\mu \pm 3\sigma$ range, attributed to natural fluctuations in the data, the results were still in alignment with the normality test.

In summary, the analysis confirms that our experimental methods are consistent, and consequently, the quality of our data is robust and reliable. Moreover, the IGK and IGL data demonstrated good stability across different patients and time points, reinforcing the reliability of our findings.

Supplementary Table 1. Characteristics of Pre and Post sample.

	Index	Pre	Post
Quality of the data	Average total cell counts	272908±166311	1361670±1075839
	Average raw data read counts(G)	1.76±1.54	3±3.55
	Average raw data Q30(%)	92.49±2.68	92.7±2.86
Stability of IGK/IGL markers	Average IGK clone counts	1385373±2012618	1406895±1722960
	Average IGL clone counts	1062789±2136410	1089539±1477219

Supplementary Table 2 Normality test results of five metrics.

	Time Point	Shapiro-Wilk		
		Statistics	df	Sig.
Total cell counts	Pre	.557	396	.000
	Post	.910	693	.000
Raw data read counts	Pre	.758	362	.000
	Post	.519	684	.000
Raw data Q30	Pre	.950	395	.000
	Post	.911	724	.000
IGK clone counts	Pre	.761	570	.000
	Post	.713	306	.000
IGL clone counts	Pre	.461	357	.000
	Post	.695	629	.000

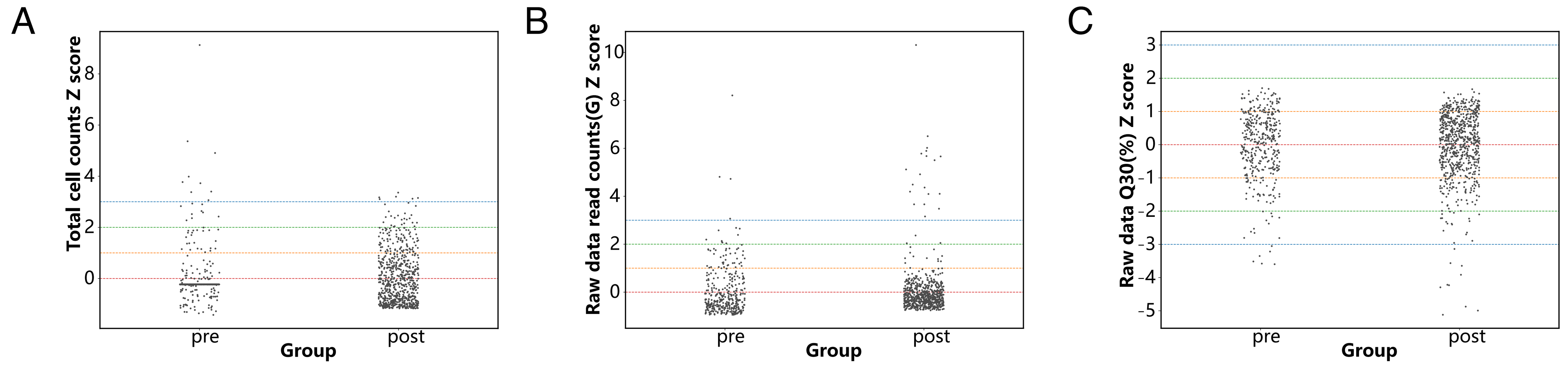

		Pre	Post	standard
Total cell counts	±1σ	83.97%	68.38%	> 68%
	±2σ	95.42%	95.83%	> 95%
	±3σ	97.71%	99.14%	≈99%

		Pre	Post	standard
Raw data read counts	μ±1σ	85.71%	95.07%	> 68%
	μ±2σ	96.36%	96.81%	> 95%
	μ±3σ	98.88%	97.24%	≈99%

		Pre	Post	standard
Raw data Q30	μ±1σ	71.03%	71.74%	> 68%
	μ±2σ	95.64%	96.30%	> 95%
	μ±3σ	98.46%	98.63%	≈99%

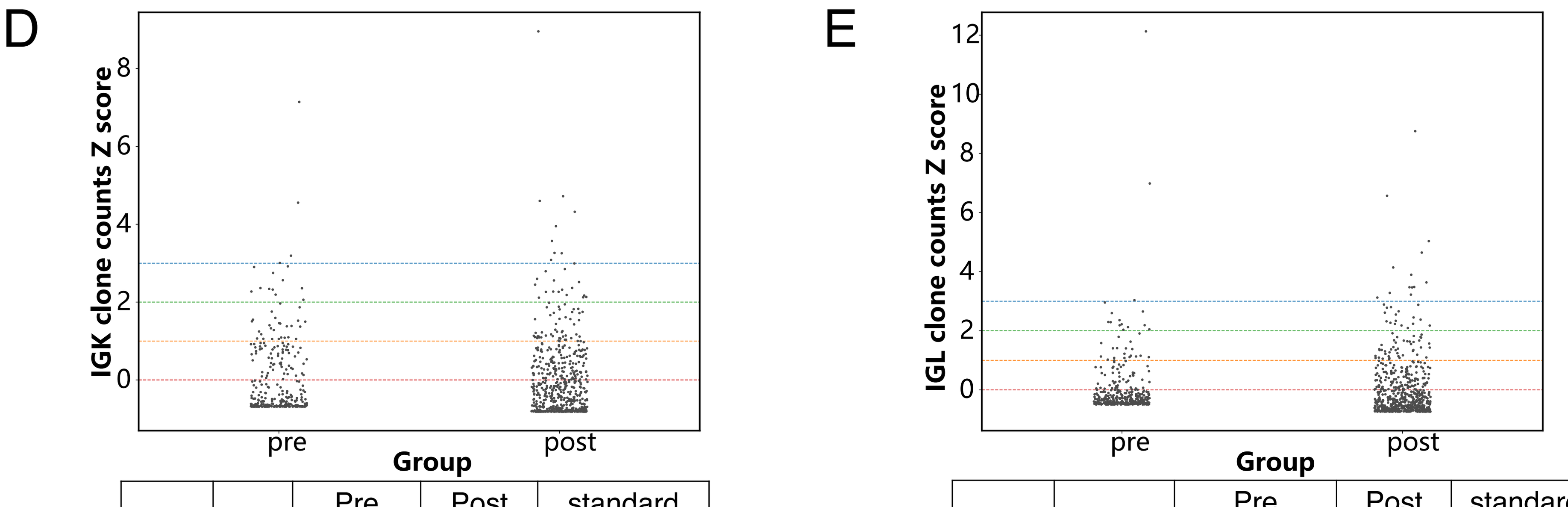

		Pre	Post	standard
IGK clone counts	μ±1σ	86.51%	87.41%	> 68%
	μ±2σ	95.07%	95.45%	> 95%
	μ±3σ	98.68%	98.43%	≈99%

		Pre	Post	standard
IGL clone counts	μ±1σ	90.70%	88.43%	> 68%
	μ±2σ	95.77%	95.25%	> 95%
	μ±3σ	99.15%	97.94%	≈99%

Supplementary Figure 1. Illustrations of the comprehensive data quality assessment. Data were performed on five metrics: Total Cell Counts (A), Raw Data Read Counts (B), Raw Data Q30 Scores (C), IGK Clone Counts (D), and IGL Clone Counts (E), using the 3-Sigma (3-σ) statistical test principle. The x-axis differentiates between Pre and Post sample groups, with individual samples denoted by black data points. The y-axis quantifies the Z-score, computed as $Z = (X - \mu) / \sigma$. Various threshold lines are color-coded: the blue dashed line marks the $\mu \pm 1\sigma$ range, the green dashed line indicates the $\mu \pm 2\sigma$ range, the orange dashed line signifies the $\mu \pm 3\sigma$ range, and the red dashed line delineates the position of the average standard deviation, denoted by μ.

Supplementary References

1. Nyhuis P, Wiendahl H-P. 3-Sigma PPC - A Holistic Approach for Managing the Logistic Performance of Production Systems. *CIRP Annals* **53**, 371-376 (2004).
2. Shannaq B, Al-Azzawi F. Three-Sigma Scale Model for Measuring Student Interest in Social Media-Effective Tool for Improving the Educational Process in the Coronavirus (Covid-19) Period. *International Journal of Advanced Science and Technology* **29**, 3597-13609 (2020).
3. Xiao H, Zhang Y, Liu X, Yin H, Liu P, Liu DC. A Rapid Ultrasound Vascular Disease Screening Method using PauTa Criterion. *Journal of Physics: Conference Series* **1769**, (2021).

2. Gating strategy for MRD detection in multiple-color flow cytometry (MFC) assay

In the gating strategy for MRD assessment, the initial step involves the exclusion of dead cells and cellular debris, based on Forward Scatter (FSC)/Side Scatter (SSC) dot plots. Additionally, cell doublets are removed by evaluating FSC Area/FSC Height plots. Next, leukemic cells are identified from the CD45/SSC dot plots based on their low SSC characteristics. B Cell Precursor (BCP) leukemic cells are then specifically gated using CD45/CD19 dot plots, determined by the expression levels of CD45 and CD19. These BCP leukemic cells are further characterized by the immunophenotype markers CD10+CD34+ and CD20-CD58+. Concurrently, we also assess other LAIP markers or cross-lineage markers such as CD13 and CD38, to identify optimal MRD markers for monitoring. As illustrated in Supplementary Figure 2A, a marker combination of CD45+CD19+CD10+CD34+CD20-CD58+ was selected at diagnosis for longitudinal MRD tracking.

During subsequent follow-up visits, the previously established marker combination and gating strategy are consistently applied for MRD evaluation. For MRD to be considered detectable, a cluster of cells must exhibit both appropriate FSC/SSC properties and an aberrant immunophenotype. The MRD percentage is calculated by dividing the number of identified leukemic cells by the total number of nucleated cells under examination. An example for MRD evaluation at the end of induction was shown in Supplementary Figure 2B.

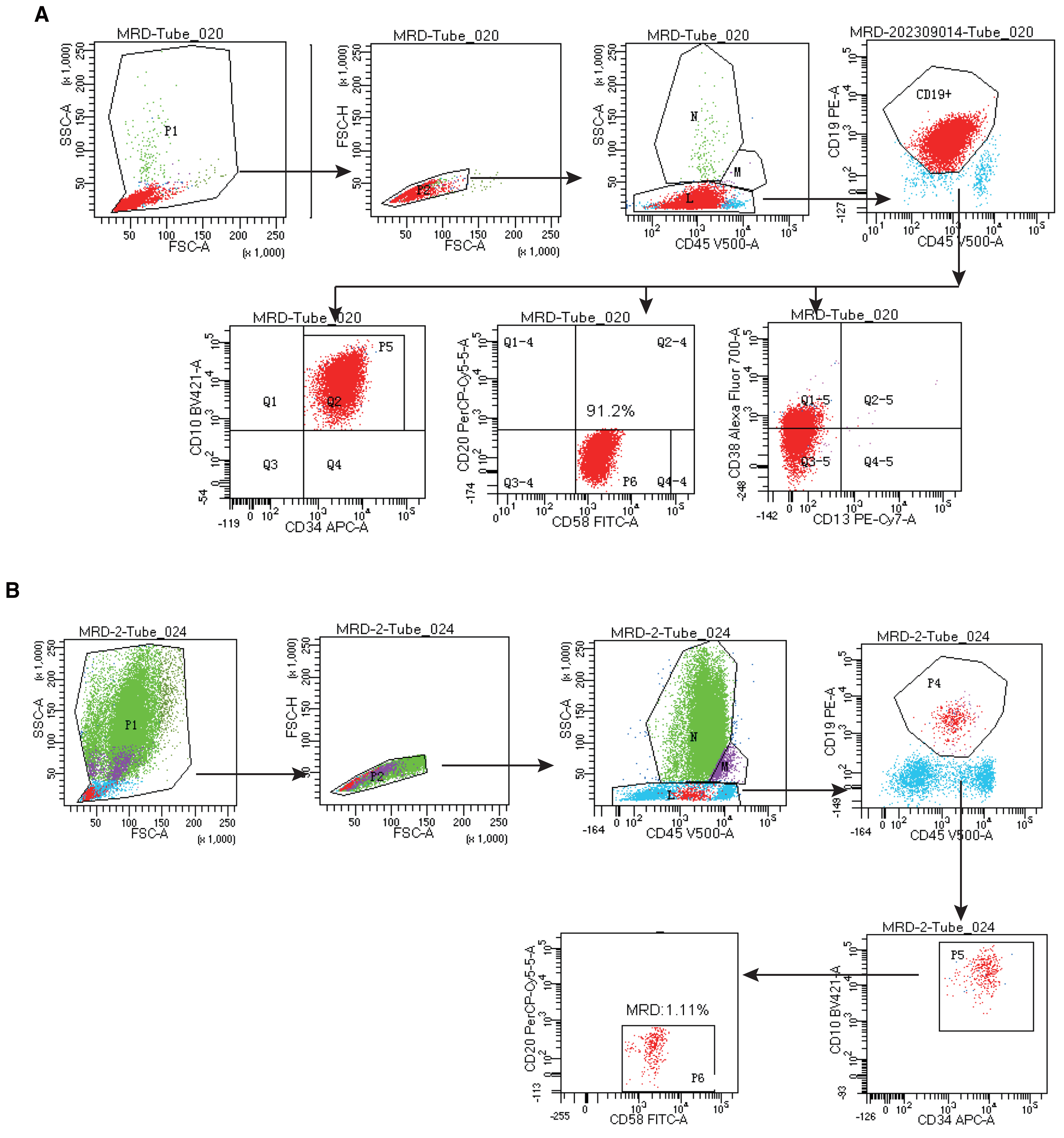

Supplementary Figure 2. Gating strategy for MRD detection in multiple-color flow cytometry (MFC) assay. The process of minimal residual disease (MRD) analysis comprises several key steps, which commence with the identification of Leukemia-Associated Immunophenotypes (LAIPs) at initial diagnosis (A). This serves as the basis for MFC-MRD assessment. Subsequent follow-up MRD evaluations adhere to a LAIP-gating strategy. Six to eight distinct antibody combinations are deployed to accurately quantify the remaining leukemic cells of interest (B).

3. ZJCH-ALL-2019 protocol

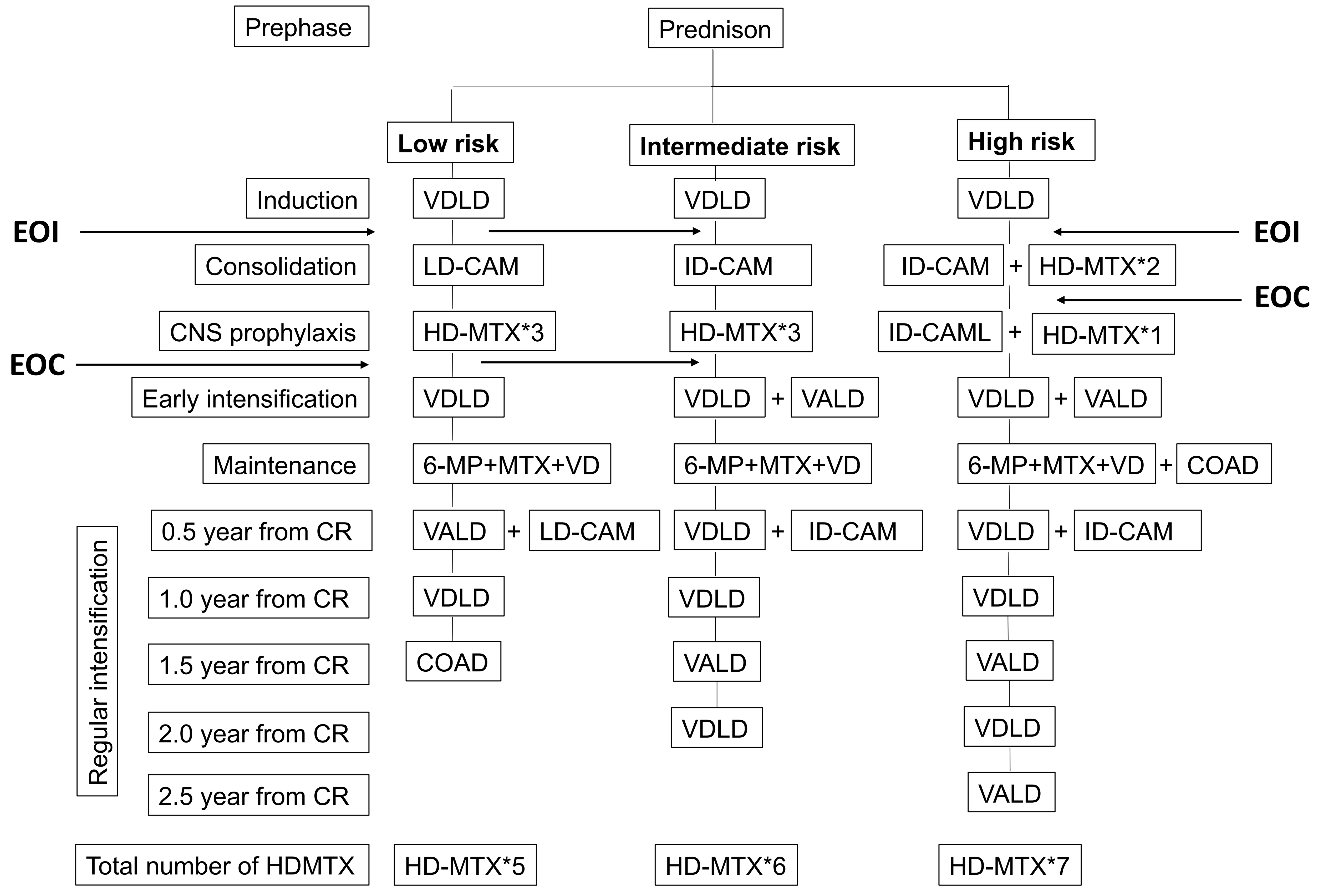

Supplementary Figure 3. ZJCH-ALL-2019 protocol

Abbreviation:

VDLD: Vincristine/Vindesine, Epirubicin, Pegaspargase, Dexamethasone;

LD-CAM (low-dose cytarabine) : Cyclophosphamide, Cytarabine, 6-mercaptopurine;

HD-MTX: High dose- methotrexate, 6-mercaptopurine

6-MP+MTX+VD: 6-mercaptopurine, Methotrexate, Vindesine, Dexamethasone

VALD: Vincristine/Vindesine, Cytarabine, Pegaspargase, Dexamethasone

COAD: Cyclophosphamide, Vincristine/Vindesine, Cytarabine, Dexamethasone

ID-CAM (intermediate-dose cytarabine): Cyclophosphamide, Cytarabine, 6-mercaptopurine;

ID-CAML: Cyclophosphamide, Cytarabine, 6-mercaptopurine, Pegaspargase

Chemotherapy regimen:

VDLD in induction: Vincristine 1.5mg/m^2 or Vindesine 3mg/m^2, d1, d8, d15, d22; Epirubicin 30mg/m^2 2-3 times; Pegaspargase 2500U/m^2/time, d8, d22; Dexamethasone 6mg/m^2,d1-d21 and tapering for one week.

LD-CAM : Cyclophosphamide 1000mg/m^2 /d, d1; Cytarabine 75mg/m^2 /d d1-d4, d8-d11,ih, 6-mercaptopurine 50mg/m^2 /d, d1-d14.

ID-CAM :Cyclophosphamide 1000mg/m^2 /d, d1; Cytarabine 1g/m^2 /time d1-d3 q12h ivgtt, 6-mercaptopurine 50mg/m^2 /d, d1-d7.

ID-CAML (intermediate-dose cytarabine): Cyclophosphamide 1000mg/m^2 /d, d1; Cytarabine 1g/m^2 /time d1-d3 q12h ivgtt, 6-mercaptopurine 50mg/m^2 /d, d1-d7, Pegaspargase 2500U/m^2/time, d4.

HD-MTX: High dose-methotrexate for low risk group: 3g/m^2/time d1, every 10 days to 2 weeks once, a total of 3 times, 6-mercaptopurine 50mg/m^2 /d, d1-d7; for intermediate and high risk: 5g/m^2/time, d1, 6-mercaptopurine 50mg/m^2 /d, d1-d7;

VDLD in intensification: Vincristine 1.5mg/m^2 or Vindesine 3mg/m^2, d1, d8; Epirubicin 30mg/m^2 d1-2; Pegaspargase 2500U/m^2/time, d1, d15; Dexamethasone 6mg/m^2 ,d1-d14.

VALD: Vincristine 1.5mg/m^2 or Vindesine 3mg/m^2, d1, d8, Cytarabine 1g/m^2 /time d1-d3 q12h, Pegaspargase 2500U/m^2/time, d4, Dexamethasone 8mg/m^2 ,d1-d8.

COAD: Cyclophosphamide 600mg/m^2 /d, d1, Vincristine 1.5mg/m^2 or Vindesine 3mg/m^2, d1, Cytarabine 100mg/m^2 /d d1-d5 q12h, Dexamethasone 6mg/m2 ,d1-d7.

Maintenance treatment: 6-MP+MTX+VD: 6-mercaptopurine 50mg/m^2 /d, d1-d21, Methotrexate 25-30mg/m^2 /d, d1, d8, d15, Vincristine 1.5mg/m^2 or Vindesine 3mg/m^2, d22, Dexamethasone 6mg/m^2 ,d22-d28.

*The number of times is determined according to the degree of risk and treatment response.

Supplementary Table 3. Risk stratification of ZJCH-ALL-2019 protocol for newly diagnosed ALL

Standard-risk group: All of the following conditions must be met.	Intermediate-risk group: Meets one or more of the following criteria:	High-risk group: Meets any one or more of the following criteria:
1. Age >= 1 year and < 10 years.<br>2. White blood cell count (WBC) < 50 × 10^9/L.<br>3. Non-CNS2, CNSL (CNS3), or/and testicular leukemia (TL).<br>4. Non-intermediate-high risk group in terms of cytogenetic and molecular biology characteristics.<br>5. Bone marrow M1 (bone marrow blasts < 5%) on day 15 of induction chemotherapy; and bone marrow M1 at end of induction (EOI).<br>6. After completion of induction therapy, there is no evidence of leukemia infiltration based on clinical and radiographic evaluations, and the mediastinal tumor lesions have disappeared.<br>7. MRD (minimal residual disease) criteria*:<br>MRD < 1 × 10^{-3} on day 15 of induction therapy, and MRD < 1 × 10^{-4} at EOI.	1. Age >= 10 years old<br>2. WBC >= 50×10^9/L<br>3. CNS2, CNSL (CNS3), and/or testicular leukemia (TL)<br>4. t(1;19), which refers to the E2A-PBX1 fusion<br>5. T-cell acute lymphoblastic leukemia (T-ALL)<br>6. iAMP217<br>7. Philadelphia chromosome-positive acute lymphoblastic leukemia (Ph+ALL)<br>8. Philadelphia-like acute lymphoblastic leukemia (Ph-like ALL)<br>9. After completion of induction therapy, evaluation shows that the mediastinal tumor mass has not disappeared but has decreased to less than one-third of the initial tumor volume, and infiltrative lesions in other sites have not completely disappeared, such as splenomegaly and lymphadenopathy.<br>10. D15 B-cell type needs to reach bone marrow M1; D15 T-cell type requires bone marrow M2 (5% < bone marrow blasts < 20%), and at EOI, the bone marrow should be M1, and it should meet the following MRD criteria.<br>11. MRD criteria:<br>(1) Induction therapy, day 15: 1×10^{-3} < MRD < 1×10^{-2}<br>(2) EOI: 1×10^{-4}< MRD < 1×10^{-2}	1. Day 15 of induction therapy: B-cell type with bone marrow M2, T-cell type with bone marrow M3 (bone marrow blasts > 25%).<br>2. bone marrow shows incomplete remission with M2 and M3 (bone marrow blasts >= 5%) at EOI.<br>3. Positive MLL gene rearrangement.<br>4. Hypodiploidy (<44 chromosomes) or DI index < 0.85.<br>5. Homozygous deletion of IKZF1 large segment.<br>6. MEF2D rearrangement.<br>7. TCF3-HLF/t(17;19) (q22;p13).<br>8. After completion of induction therapy, on evaluation between days 29-33, the mediastinal tumor lesions have not decreased to less than one-third of the original tumor volume.<br>9. Immunophenotype is Early T-cell precursor (ETP).<br>10. MRD criteria:<br>(1) B-cell type: MRD >= 1 × 10^{-2} on day 15 of induction therapy or MRD >= 1 × 10^{-2} at EOI.<br>(2) T-cell type: MRD >= 1 × 10^{-2} at EOI.

*, MRD used for risk stratification in this protocol was detected by multiparameter flow cytometry or quantitative polymerase chain reaction, but not next-generation sequencing.
