# Supplementary material for: Minimal residual disease detection by next-generation sequencing of different immunoglobulin gene rearrangements in pediatric B-ALL
Source: Nat Commun. 2023 Nov 17;14:7468. doi: 10.1038/s41467-023-43171-9 (PMC10656538; doi:10.1038/s41467-023-43171-9)
Supplement: Supplementary file 3 — Reporting Summary [file 41467_2023_43171_MOESM3_ESM.pdf]

nature portfolio

Corresponding author(s): Xiao-Jun Xu

Last updated by author(s): Jul 22, 2023

Reporting Summary

Nature Portfolio wishes to improve the reproducibility of the work that we publish. This form provides structure for consistency and transparency in reporting. For further information on Nature Portfolio policies, see our Editorial Policies and the Editorial Policy Checklist.

Statistics

For all statistical analyses, confirm that the following items are present in the figure legend, table legend, main text, or Methods section.

n/a	Confirmed	
☐	☒	The exact sample size (*n*) for each experimental group/condition, given as a discrete number and unit of measurement
☒	☐	A statement on whether measurements were taken from distinct samples or whether the same sample was measured repeatedly
☐	☒	The statistical test(s) used AND whether they are one- or two-sided<br>*Only common tests should be described solely by name; describe more complex techniques in the Methods section.*
☒	☐	A description of all covariates tested
☐	☒	A description of any assumptions or corrections, such as tests of normality and adjustment for multiple comparisons
☐	☒	A full description of the statistical parameters including central tendency (e.g. means) or other basic estimates (e.g. regression coefficient) AND variation (e.g. standard deviation) or associated estimates of uncertainty (e.g. confidence intervals)
☐	☒	For null hypothesis testing, the test statistic (e.g. *F*, *t*, *r*) with confidence intervals, effect sizes, degrees of freedom and *P* value noted<br>*Give P values as exact values whenever suitable.*
☒	☐	For Bayesian analysis, information on the choice of priors and Markov chain Monte Carlo settings
☐	☒	For hierarchical and complex designs, identification of the appropriate level for tests and full reporting of outcomes
☐	☒	Estimates of effect sizes (e.g. Cohen's *d*, Pearson's *r*), indicating how they were calculated

Our web collection on statistics for biologists contains articles on many of the points above.

Software and code

Policy information about availability of computer code

Data collection	The data in this study was collected using Microsoft Excel (version 16.0.14332.20501) and SPSS software (version 23.0.0.0).
Data analysis	Statistical analysis was performed in Graphpad Prism 9.0.0 and SPSS software (version 23.0.0.0). Data visualization was performed in R version 4.0.3 using the following packages: ggplot2, survival, survminer and networkD3 package and GraphPad Prism 9.0.0. Custom code is available via Github at https://github.com/pediatric-B-ALL-NGS/pediatric-B-ALL-NGS.

For manuscripts utilizing custom algorithms or software that are central to the research but not yet described in published literature, software must be made available to editors and reviewers. We strongly encourage code deposition in a community repository (e.g. GitHub). See the Nature Portfolio guidelines for submitting code & software for further information.

Data

Policy information about availability of data

All manuscripts must include a data availability statement. This statement should provide the following information, where applicable:

- Accession codes, unique identifiers, or web links for publicly available datasets
- A description of any restrictions on data availability
- For clinical datasets or third party data, please ensure that the statement adheres to our policy

The export of genetic information (raw sequencing data) and materials relevant to this work have been approved by the Ministry of Science and Technology of China. The raw sequencing data of this research are available under restricted access at the Genome Sequence Archive (GSA) for Human, Project ID HRA005729, and can be found at https://ngdc.cncb.ac.cn/gsa-human/browse/HRA005729. The reason for the restricted access to the data is to protect individual genetic

information, to ensure that researchers comply with the corresponding regulations when using the data, and to prevent its use for commercial purposes. Each application for data access will be reviewed individually; access to the data is intended for scientific research purposes and is available predominantly to researchers affiliated with an accredited institution, who can demonstrate a legitimate scientific purpose. Applicants must submit a detailed research plan to the corresponding author, clarifying the objectives of their proposed study and affirming their commitment to non-commercial use of the data. They must also agree not to redistribute the data. Whether a request is approved will depend on the consistency of the proposed research with ethical guidelines and data usage agreements. Once access is granted, the data will be available to researchers for the duration agreed upon within the data access agreement, typically for no longer than one year, with the possibility of extension upon request. The commitment is to respond to access requests within 10 business days. We are committed to facilitating prompt access to the data after receiving complete and adequately justified requests.
The remaining data pertinent to this study are included within the Article, Supplementary Information, or the Source Data file and are freely accessible without restrictions.

Research involving human participants, their data, or biological material

Policy information about studies with human participants or human data. See also policy information about sex, gender (identity/presentation), and sexual orientation and race, ethnicity and racism.

Reporting on sex and gender	Our study does not specifically apply to a particular sex or gender. We did not consider sex and gender in the study design. Sex and/or gender information was determined based on self-reporting.
Reporting on race, ethnicity, or other socially relevant groupings	We did not use any socially constructed or socially relevant categorization variables in our study. Therefore, there was no need for definitions, classification methods, or controlling for confounding variables related to such variables.
Population characteristics	The population characteristics of our human research participants included individuals below the age of 18, with an average age of 4.4 years. They were diagnosed with acute lymphoblastic leukemia and were undergoing treatment following the ZJCH-ALL-2019 protocol.
Recruitment	Participants were recruited for this study from November 2018 to June 2022 at the Children's Hospital of Zhejiang University School of Medicine. Children <=18 years with newly diagnosed B-ALL who undergone NGS of B-cell receptors were included in this study. Patients who were not tested NGS-MRD at both the end of induction and end of consolidation were excluded from the analysis. Patients with extramedullary leukemia at diagnosis were excluded.
Ethics oversight	This study was approved by the institutional review board of Children's Hospital, Zhejiang University School of Medicine.

Note that full information on the approval of the study protocol must also be provided in the manuscript.

Field-specific reporting

Please select the one below that is the best fit for your research. If you are not sure, read the appropriate sections before making your selection.

☒ Life sciences ☐ Behavioural & social sciences ☐ Ecological, evolutionary & environmental sciences

For a reference copy of the document with all sections, see nature.com/documents/nr-reporting-summary-flat.pdf

Life sciences study design

All studies must disclose on these points even when the disclosure is negative.

Sample size	No sample size was not specifically calculated before this study. This was a retrospective study of all eligible children with ALL between November 2018 and June 2022. Sample size was not predetermined based on statistical methods, but was chosen on the basis of prior studies that showed significant effects with similar or less sample sizes (Svaton M, et al. Blood 141, 529-533 (2023); Pulsipher MA, et al. Blood 125, 3501-3508 (2015)).
Data exclusions	Five patients who had trackable Ig rearrangements but did not test NGS-MRD at both the end of induction and end of consolidation were excluded. Three patients with extramedullary leukemia at diagnosis were excluded.
Replication	The study did not involve replication attempts to verify the findings.
Randomization	The study was observational study and randomization was not needed.
Blinding	Blinding was not relevant to this study as there were no prior assumptions about experimental outcomes and patients were not divided into experimental and control arms.

Reporting for specific materials, systems and methods

We require information from authors about some types of materials, experimental systems and methods used in many studies. Here, indicate whether each material, system or method listed is relevant to your study. If you are not sure if a list item applies to your research, read the appropriate section before selecting a response.

Materials & experimental systems

n/a	Involved in the study	
x		Antibodies
x		Eukaryotic cell lines
x		Palaeontology and archaeology
x		Animals and other organisms
	x	Clinical data
x		Dual use research of concern
x		Plants

Methods

n/a	Involved in the study	
x		ChIP-seq
x		Flow cytometry
x		MRI-based neuroimaging

Clinical data

Policy information about clinical studies

All manuscripts should comply with the ICMJEguidelines for publication of clinical research and a completedCONSORT checklist must be included with all submissions.

Clinical trial registration	NCT05973032
Study protocol	ZJCH-ALL-2019 protocol detailed in the supplementary file
Data collection	The enrollment of patients was between November 2018 and June 2022, and the patients were followed up until August 20, 2022. Data collection occurred between August 2022 and December 2022 at the Children's Hospital of Zhejiang University School of Medicine.
Outcomes	Primary outcome was event free survival. Death during induction, abandonment before complete remission, death in continuous complete remission, relapse, and secondary malignancies were considered as events in the calculation of EFS probability. The secondary outcome was Ig clonal clearance status which was evaluated at end of induction (EOI) and end of consolidation (EOC).
